# Methane-Linked Mechanisms of Electron Uptake from Cathodes by Methanosarcina barkeri

**DOI:** 10.1128/mBio.02448-18

**Published:** 2019-03-12

**Authors:** Annette R. Rowe, Shuai Xu, Emily Gardel, Arpita Bose, Peter Girguis, Jan P. Amend, Mohamed Y. El-Naggar

**Affiliations:** a Department of Biological Science, University of Cincinnati, Cincinnati, Ohio, USA; b Department of Physics and Astronomy, University of Southern California, Los Angeles, California, USA; c Department of Earth Sciences, University of Southern California, Los Angeles, California, USA; d Department of Organismal and Evolutionary Biology, Harvard University, Cambridge, Massachusetts, USA; e Department of Biological Sciences, University of Southern California, Los Angeles, California, USA; f Department of Chemistry, University of Southern California, Los Angeles, California, USA; University of California, Irvine

**Keywords:** archaea, *Methanosarcina*, bioelectrochemistry, cathode, electrosynthesis, methanogenesis, methanogens, syntrophs

## Abstract

The *Methanosarcinales*, a lineage of cytochrome-containing methanogens, have recently been proposed to participate in direct extracellular electron transfer interactions within syntrophic communities. To shed light on this phenomenon, we applied electrochemical techniques to measure electron uptake from cathodes by Methanosarcina barkeri, which is an important model organism that is genetically tractable and utilizes a wide range of substrates for methanogenesis. Here, we confirm the ability of M. barkeri to perform electron uptake from cathodes and show that this cathodic current is linked to quantitative increases in methane production. The underlying mechanisms we identified include, but are not limited to, a recently proposed association between cathodes and methanogen-derived extracellular enzymes (e.g., hydrogenases) that can facilitate current generation through the formation of reduced and diffusible methanogenic substrates (e.g., hydrogen). However, after minimizing the contributions of such extracellular enzymes and using a mutant lacking hydrogenases, we observe a lower-potential hydrogen-independent pathway that facilitates cathodic activity coupled to methane production in M. barkeri. Our electrochemical measurements of wild-type and mutant strains point to a novel and hydrogenase-free mode of electron uptake with a potential near −484 mV versus standard hydrogen electrode (SHE) (over 100 mV more reduced than the observed hydrogenase midpoint potential under these conditions). These results suggest that M. barkeri can perform multiple modes (hydrogenase-mediated and free extracellular enzyme-independent modes) of electrode interactions on cathodes, including a mechanism pointing to a direct interaction, which has significant applied and ecological implications.

## Abstract (precis)

Methanogenic archaea are of fundamental applied and environmental relevance. This is largely due to their activities in a wide range of anaerobic environments, generating gaseous reduced carbon that can be utilized as a fuel source. While the bioenergetics of a wide variety of methanogens have been well studied with respect to soluble substrates, a mechanistic understanding of their interaction with solid-phase redox-active compounds is limited. This work provides insight into solid-phase redox interactions in Methanosarcina spp. using electrochemical methods. We highlight a previously undescribed mode of electron uptake from cathodes that is potentially informative of direct interspecies electron transfer interactions in the *Methanosarcinales*.

## INTRODUCTION

The ability of microbes to generate methane, a major component of natural gas and biogas from anaerobic digestion, as well as a powerful greenhouse gas, has important technological and environmental implications. The formation of methane through the reduction of carbon dioxide (CO_2_) is predominantly mediated by members of the Euryarchaeota (1). The energy captured during this process reflects one of life’s lowest-energy-yielding metabolisms (2). Hydrogen (H_2_), the predominant electron donor for methanogenesis, is strikingly close in redox potential to CO_2_ (the primary terminal electron acceptor) under standard conditions, which illustrates the relatively slim energetic yields often available to methanogens (3). As such, these organisms employ a variety of ecologic and biochemical strategies (some newly and/or poorly understood) in order to persist in the wide range of anoxic environments, including some of the deepest and hottest ecosystems on Earth (4, 5).

One or more steps in the methanogenesis pathway coupling CO_2_ reduction from H_2_ (and in some cases formate) oxidation can be endergonic (energy consuming) under physiologic conditions (6). The initial reduction step, from CO_2_ to a formylmethanofuran intermediate (6), requires ferredoxin, an iron-sulfur electron carrier with a lower redox potential than H_2_ in most biological contexts (1). Two strategies are known for (re)generating reduced ferredoxin in methanogens (7). The first strategy, utilized by the lineage that does not contain cytochromes, couples the first ferredoxin-requiring step (energetically unfavorable), with the final reduction of a methyl group bound to coenzyme M to produce methane and regenerate the coenzyme M-coenzyme B intermediate (energetically favorable) (8, 9). This occurs via a flavin-mediated electron-bifurcating enzyme complex (8, 9). The second strategy, utilized by methanogenic clades that contain cytochromes, employs an energy-converting hydrogenase (Ech) that utilizes a cellular ion motive force to drive the energetically uphill reduction of ferredoxin from H_2_ (7). The biochemical differences represented in these two strategies underlie the ecological trade-offs between respiration rate (allowing faster substrate utilization) and energetic yield (maximizing the efficiency of energy capture), which often dictate the differential success of these groups under various competitive environmental conditions.

In many environmental systems, hydrogenotrophic methanogens also face energy challenges with respect to electron donor limitation, given that environmental H_2_ concentrations are commonly near or below the energetic threshold for methanogenesis (3, 6). It has been suggested that H_2_ is often of syntrophic origin in methanogenic environments. Syntrophic interactions involve a metabolic intermediate (i.e., hydrogen), generated via fermentation at low hydrogen partial pressures by one organism and being consumed by a partnering microbe (i.e., methanogen) (10). The energetic favorability of syntrophic fermentations is stabilized by the subsequent consumption of fermentation intermediates by the syntrophic partners, thereby maintaining favorable environmental conditions. The first such example was the coculture of an ethanol-fermenting bacterium and a hydrogenotrophic methanogen (11). Though this metabolic strategy is thought to dominate in many anoxic environments, the prevalence of hydrogen and/or formate as a metabolic intermediate has been widely debated (12, 13). It has proved challenging to match the known rates of diffusion for these intermediates with the necessary concentrations that would reflect energetic favorability for both the fermenter and methanogen syntrophic partners. Several studies have suggested that the rates of diffusion are too low at syntrophically relevant concentrations to account for the rates of methanogenesis that are observed in many environmental systems (14). Put simply, a faster means of electron transfer is likely necessary to explain the measured rates.

A previously demonstrated coculture of the ethanol-fermenting Geobacter metallireducens and methane-producing Methanosarcina barkeri highlighted the potential for a hydrogen-free and formate-free mode of interspecies electron transfer (15). The ability of G. metallireducens to perform direct extracellular electron transfer (EET) to solid surfaces is well known (16). Since mutants impaired in this direct EET were incapable of forming viable ethanol-consuming methane-generating consortia (15), it was suggested that these cocultures share electrons through direct interspecies electron transfer (DIET) (17). However, while the mechanistic basis of direct outward EET from Geobacter species is well studied, there is currently no proposed mechanism for such a direct inward EET mechanism into the methanogenic partners.

To address this knowledge gap, we investigated the potential of M. barkeri to interact with solid-phase electron sources (i.e., cathodes) as a surrogate for obtaining electrons from a syntrophic partner. To date, the only known mode of cathodic electron uptake by methanogens is catalyzed by cell-derived free enzymes (predominantly hydrogenases) that can attach to electrodes (18). In that case, methane is generated from electrochemically produced electron donors, such as H_2_ or formate (18). Notably, these findings were obtained with Methanococcus maripaludis, an organism in the clade of methanogens without cytochromes. We hypothesized that a previously unknown mode of electron transfer may be present in cytochrome-containing methanogens and that this mode may be distinguished electrochemically. Here, we apply electrochemical techniques on wild-type and a hydrogenase deletion mutant of M. barkeri, a cytochrome-containing methanogen previously shown to generate methane in consortia with G. metallireducens, to investigate the potential for direct electron uptake in these microbes. This has potentially important implication for understanding both the ecologic trade-offs between direct and hydrogen-based syntrophic partnerships, as well as in the potential utility of these organisms in bioelectrode technologies.

## RESULTS

### M. barkeri generates methane in electrochemical cells with poised potentials lower than −400 mV versus SHE.

**FIG 1:**
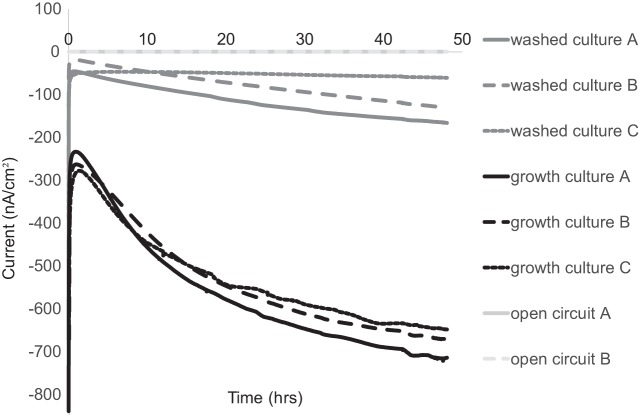
Electron uptake occurs in *M. barkeri* with and without growth medium present at −450 mV. Comparison of current consumption in *M. barkeri* cells on poised electrodes. This includes experiments where (i) the growth medium was removed (washed culture), (ii) cells were added along with the growth medium (growth culture); or (iii) cells were washed to remove growth medium but no potential was poised on the electrode (open circuit).

We tested both methane production and electron uptake (cathodic current) by M. barkeri cell cultures in 3-electrode H-cell electrochemical reactors, with the working carbon cloth electrode poised between −400 mV and −500 mV under two different culture conditions. The first condition consisted of cells in their growth or spent medium (growth cultures), and the second condition represented washed cells where the pregrown cultures were centrifuged and resuspended in fresh basal medium lacking electron donor, reductants, vitamins, and minerals (washed cultures). This second condition was chosen to mitigate the potential effects of the growth or spent medium containing free enzymes (e.g., hydrogenases) capable of attaching to carbon electrodes and potentially masking cell-electrode interactions with enzyme-electrode reactions (as described in reference 18). Compared to open circuit controls, M. barkeri cell cultures demonstrated increased methane production and yielded cathodic currents (Table 1). Both current and methane production were larger in preparations that included spent medium (i.e., growth cultures), but both poised potential electrode experiments (i.e., growth and washed cultures) produced more methane than did the open circuit controls (Table 1 and Fig. 1). Notably, the coulombic efficiencies (percentage of electrons that could be accounted for in the methane produced) were significantly larger in washed culture incubations than in the growth cultures (Table 1). As previously suggested (18), this may be due to the influence of free hydrogenases in the spent medium interacting with electrodes, resulting in a hydrogen pool that accounts for a portion of the coulombs drawn from electrode but not converted to methane. To further investigate this, we performed cyclic voltammetry (CV) to determine the patterns of electron uptake relative to the redox potentials in the aforementioned experiments.

**TABLE 1:** Methane generated and current consumption measured in wild-type *M. barkeri* experiments per experiment depicted in Fig. 1^*a*^

Culture	Measured methane (eeq total)	Predicted based on current (eeq total)	Avg coulombic efficiency (%)
Washed cultures	18.9 ± 3.3	18.3 ± 7.3	102 ± 44
Growth cultures	104.0 ± 15.7	120.3 ± 35.5	86 ± 28
Open circuit	7.5 ± 5.1		

a Each value represents the average of the results from five experimental data sets plus or minus the standard deviations. Coulombic efficiency was calculated from the mean data.

### Multiple electron uptake features observed with cyclic voltammetry.

**FIG 2:**
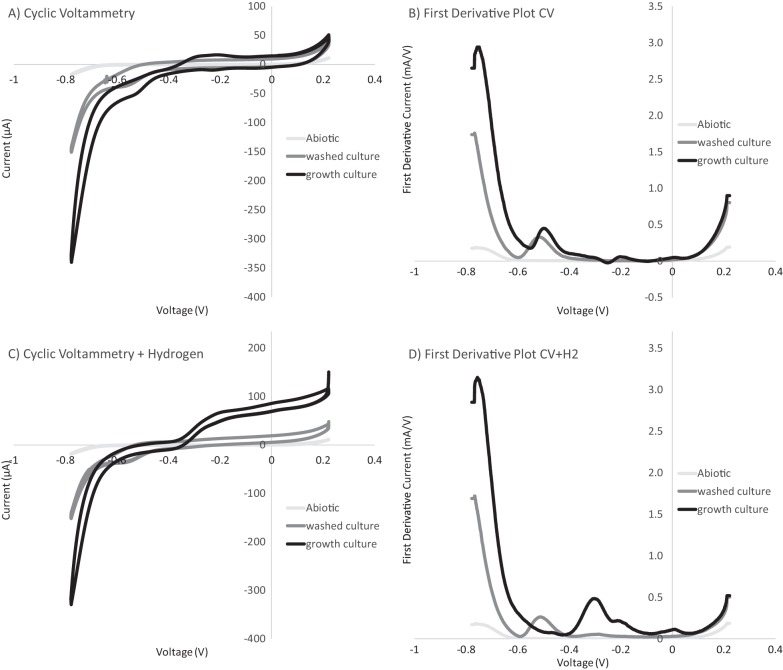
Differences observed in electron uptake features between washed cultures and growth culture experiments. (A) Cyclic voltammetry (1-mV/s scan rate over a range of −800 to 200 mV) comparing voltage and current relationships between poised potential (−450 mV) experiments (shown in Fig. 1). With the exception of abiotic controls (run preexperiment), all cyclic voltammetry was performed postchronoamperometry. (B) First derivative plot of the cathode sweep (positive voltage to negative voltage) for each cyclic voltammetry (CV) comparing peaks of electron uptake features observed. (C) Replacing the headspace with a hydrogen and carbon dioxide gas mix (80% and 20%, respectively) demonstrates that some features are sensitive to hydrogen concentration. (D) First derivative plot of the cathode sweep (positive voltage to negative voltage) for each hydrogen addition CV demonstrates the changes in features sensitive to hydrogen.

Cyclic voltammograms demonstrated two distinct catalytic features observed over the described experimental conditions. One catalytic feature was observed in both growth culture and washed culture experiments. A second more positive catalytic wave was observed only under the growth culture incubations (which included putative free enzymes) (Fig. 2A). The shared and predominant cathodic feature, as demonstrated by the first derivatives of the cathodic sweep of cyclic voltammograms, peaked at −473 mV to −484 mV versus SHE for growth cultures and washed cultures, respectively (Fig. 2B). In the growth culture experiments, the first derivative analysis revealed other subtle features at higher redox potentials (−182 ± 5.6 mV and −279 ± 2.3 mV versus SHE, Fig. 2B). The −279-mV feature is consistent with the second catalytic feature observed and proximal to the redox potential for H_2_ production at pH 6.5 to 7 in this high-salt system (range, 380 to 300 mV, depending on ionic strength). Notably, the corresponding feature observed in the first derivative of the anodic sweep (−272 ± 5.1 mV versus SHE; see Fig. S1A in the supplemental material), and the small offset between the anodic and cathodic peaks is consistent with the activity of an electrode-attached enzyme (19, 20). This is in contrast to the ∼100-mV separation measured for the shared electron uptake feature (−484 ± 9.4 mV and −579 ± 13.3 mV versus SHE for the anodic and cathodic sweeps, respectively) (Fig. S1B), which is consistent with previous observations of the more distant cell-electrode interactions (21–23). The −182-mV diffusion-limited electrochemical feature observed is consistent with the redox indicator added to the growth medium, resazurin, which has been observed electrochemically in growth medium without cells but not the basal medium used in electrochemical experiments.

10.1128/mBio.02448-18.1 FIG S1 Dominant electron uptake feature in spent medium plus cells experiments resemble an electrode-bound enzyme process. (A and B) First derivative plots of anodic (negative to positive) and cathodic (positive to negative) sweeps from cyclic voltammetry of growth culture (A) and washed culture (B) experiments. Cyclic voltammetry data are shown in Fig. 2. Download FIG S1, DOCX file, 0.2 MB. Copyright © 2019 Rowe et al. 2019 Rowe et al. This content is distributed under the terms of the Creative Commons Attribution 4.0 International license.

To determine if one or both CV features are indicative of hydrogenase activity, the same electrochemical reactors were purged with an 80%/20% H_2_/CO_2_ gas mix to assess the impact of increasing H_2_ concentration. Given that hydrogenases are reversible enzymes, we expected that hydrogen would provide a substrate for oxidation by the enzymes, generating anodic current and inhibiting electron uptake. As predicted, a dramatic anodic catalytic wave was observed at the predicted hydrogenase redox potential in growth cultures when H_2_ concentrations were around 0.6 mM (Fig. 2C). This wave is consistent with hydrogenase-driven H_2_ oxidation at redox potentials above −380 mV (onset potential observed in the anodic sweep of CV from Fig. 2C). Neither catalytic activity (Fig. 2C) nor a shift in redox potential of the predominant −484-mV feature (Fig. 2D) was significantly altered by increasing H_2_ in the washed cell experiments. Conversely, hydrogen greatly affected the putative hydrogenase feature in the growth culture experiments, which dramatically increased and dominated both the CV and first derivative plots of the cathode sweep in these experiments (Fig. 2C and D). Taken collectively, the impact of different cell preparations and H_2_ addition suggests the presence of multiple electron uptake mechanisms in these M. barkeri electrochemical systems, including both hydrogen-dependent (hydrogenase-mediated) and hydrogen-independent (non-hydrogenase-mediated) mechanisms.

### Current generation observed in M. barkeri hydrogenase deletion mutant with electrodes poised below −400 mV.

**FIG 3:**
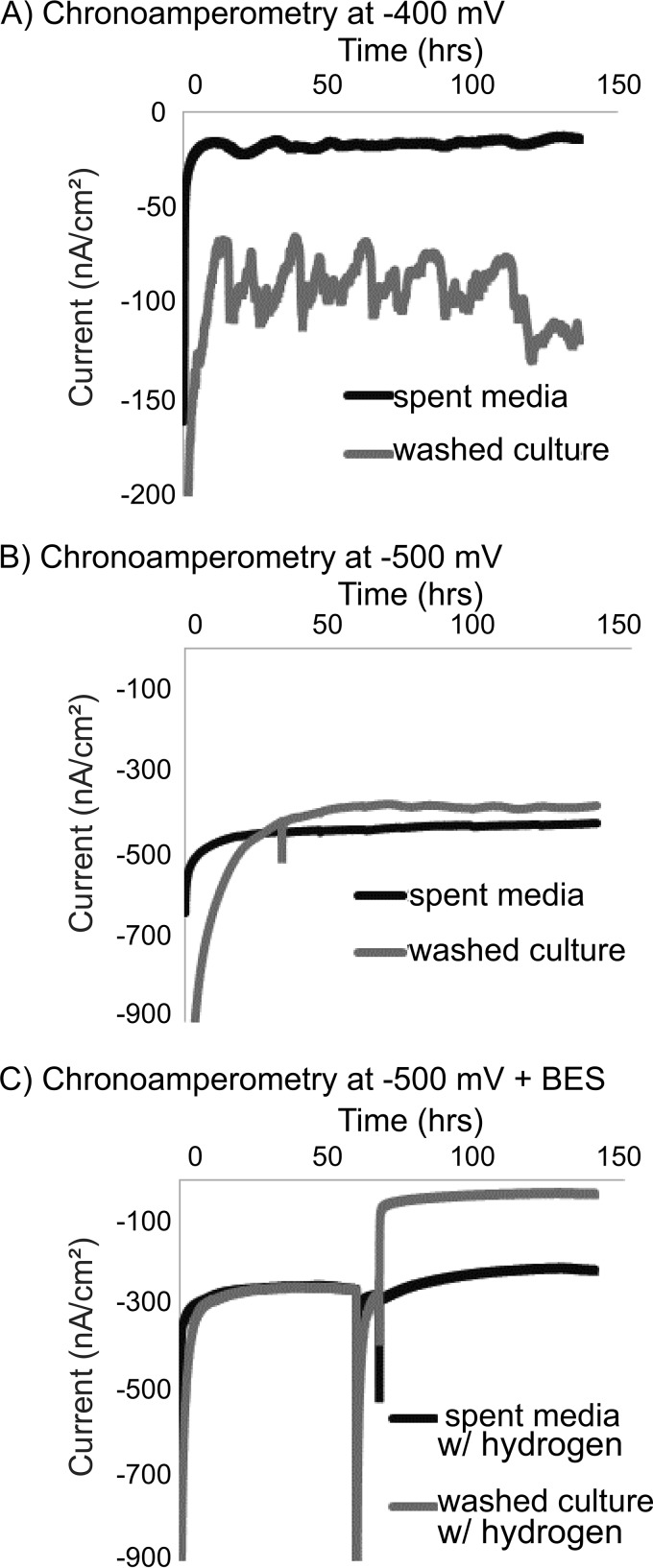
Current consumption occurs in a hydrogenase deletion mutant (Δ*hyd*) across a range of −400 to −500 mV and is inhibited by BES. Representative current profiles for the *M. barkeri* mutant (Δ*hyd*) (described in reference 28), including experiments with cells (washed culture) and the cell growth medium only (spent media) at both −400 mV (A) and −500 mV (B). Changing the hydrogen concentration to include an 80%/20% mix of hydrogen and carbon dioxide did not alter the current consumption, though the addition of BES at 18 h demonstrated inhibition in the reactors containing cells (C).

To confirm that a non-hydrogenase-mediated mechanism of electron uptake can be utilized for methane formation, electrochemical experiments were performed using a hydrogenase deletion mutant of M. barkeri (24). This mutant lacks the genes for all three types of hydrogenase present in M. barkeri, the ferredoxin-dependent energy-converting hydrogenase (Ech), the cytoplasmic F_420_-dependent hydrogenase (Frh), and the periplasmic methanophenazine-dependent hydrogenase (Vht). Furthermore, this mutant does not grow with hydrogen as an electron donor or via acetate disproportionation. However, this strain is capable of growth via methanol disproportionation (24). Cathodic currents (i.e., electron uptake) were generated with this mutant in three-electrode systems, with the working electrodes poised from −400 mV to −500 mV (Fig. 3A and B). The coulombic efficiencies of current linked to methane production for washed preparations of the hydrogenase mutant were comparable (120% ± 72%) to those observed in the wild-type washed culture preparations (Table 1).

Cathodic current generation in preparations lacking cells (spent medium) of the hydrogenase deletion mutant was observed to be consistently less than that in the washed cell experiments (Fig. 3A and B). Additionally, no methane or hydrogen production was observed in the spent medium-only experiments from the hydrogenase deletion mutant. The lack of hydrogenases, and consequently the lack of hydrogen formation in these systems, was further confirmed by the observation that changing the headspace composition of these reactors to a high hydrogen concentration (80%/20% H_2_/CO_2_) did not impact cathodic current production in the spent medium or washed cell incubations of the hydrogenase mutant (Fig. 3C, first 10 h). To confirm that the electron uptake currents observed in the washed cell experiments were the result of methanogen activity on electrodes, we used 2-bromoethanesulphonate (BES) as an inhibitor of methanogenesis. The addition of 7 mM BES resulted in a dramatic decrease in electron uptake in the washed cell incubation of the hydrogenase deletion mutant but not in the spent medium incubation from the deletion mutant (Fig. 3C). This further supports the idea that the observed cathodic electron uptake feature is coupled to methanogenesis.

### Hydrogenase-independent electron uptake feature observed in an M. barkeri hydrogenase deletion mutant.

**FIG 4:**
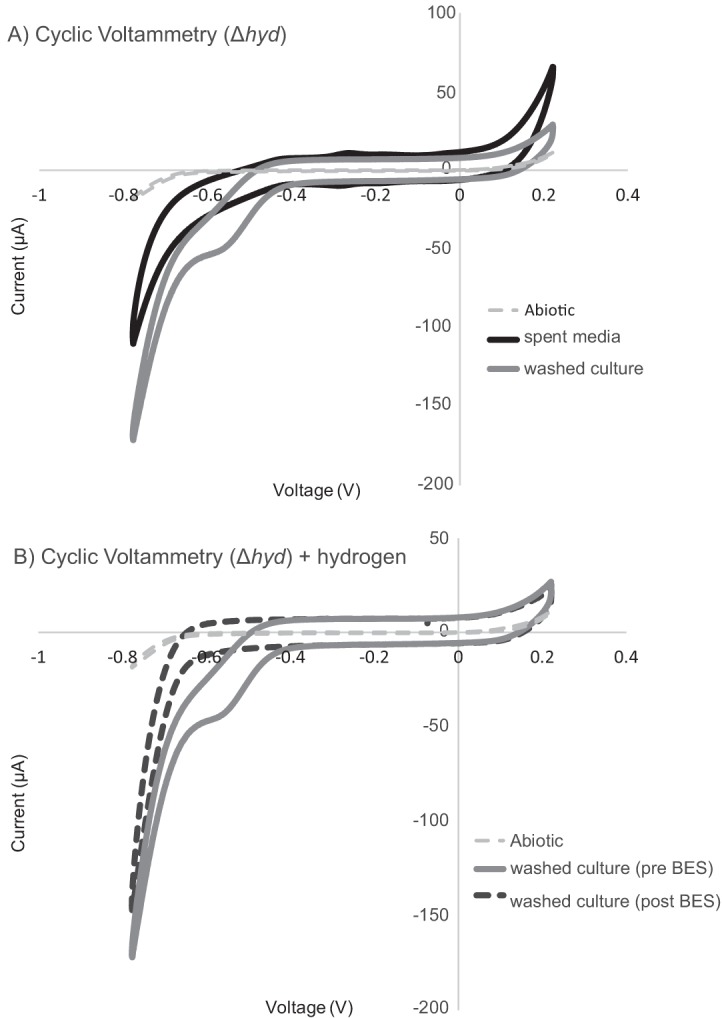
Electron uptake profile of hydrogenase deletion mutant (Δ*hyd*) is similar to *M. barkeri* wild-type washed culture experiments and is diminished in the presence of BES. (A) Cyclic voltammetry (1-mV/s scan rate over a range of −800 to 200 mV) showing the current-voltage relationship for washed cultures of the Δ*hyd* mutant compared with spent medium from the Δ*hyd* mutant and blank medium abiotic controls. With the exception of abiotic controls (run preexperiment), all cyclic voltammetry was performed postchronoamperometry (shown in Fig. 3). (B) Comparing washed culture experiment cyclic voltammetry, hydrogen addition (headspace of 80%/20% H_2_/CO_2_) results in no change in the electron uptake features observed; however, the addition of 7 mM BES abolishes the electron uptake feature.

Cyclic voltammetry in washed cultures of the M. barkeri hydrogenase deletion mutant demonstrated a single low-potential electron uptake feature consistent with the feature previously observed for wild-type M. barkeri cultures at −484 mV versus SHE (Fig. 2 and 4A). The deletion mutant experiments were compared with cell-free or spent medium conditions, where cell cultures were removed via centrifugation. Notably, this feature was not observed in the spent medium experiments, supporting the idea that this feature is cell associated rather than extracellular enzyme based (Fig. 4A). Cyclic voltammetry confirms that electron uptake in both the spent medium (Fig. S2) and washed cell (Fig. 4B) experiments was not altered by the addition of 80%/20% H_2_/CO_2_, suggesting that hydrogen formation is not occurring in these reactors nor is it affecting current and methane production. This supports the hydrogen and hydrogenase-independent nature of the low-potential electron uptake feature observed in M. barkeri. The lack of a similar feature in the spent medium experiments also supports the idea that the electron uptake feature observed is cell associated rather than due to a free extracellular enzyme.

10.1128/mBio.02448-18.2 FIG S2 Spent medium of hydrogenase deletion mutant shows little to no change with the addition of hydrogen and BES. Cyclic voltammetry (1-mV/s scan rate over a range of −800 to 200 mV) showing the current-voltage relationship for spent medium-only controls under experimental conditions with a nitrogen and carbon dioxide atmosphere (80%/20% N_2_/CO_2_), a hydrogen and carbon dioxide atmosphere (80%/20% H_2_/CO_2_), and with 7 mM BES added. Download FIG S2, DOCX file, 0.1 MB. Copyright © 2019 Rowe et al. 2019 Rowe et al. This content is distributed under the terms of the Creative Commons Attribution 4.0 International license.

With the addition of BES to washed cells, the formerly observed electron uptake feature is no longer present (Fig. 4B). However, BES addition did not alter electron uptake in the cell-free spent medium controls (Fig. S2). This suggests a physiologic linkage between the low-potential electron uptake and generation of methane by M. barkeri. Inhibition by BES was rapid, occurring in 10 to 15 min (Fig. 3C). In other methanogenic electrode systems, a slow onset to BES inhibitions was observed, as was a corresponding spike in hydrogen (25). In this alternate case, the inhibition of current was likely linked to product inhibition or specifically the accumulation of hydrogen-inhibiting hydrogenase activity. The rate of this inhibition (previously observed to occur at slower time scales of 10+ h [25]), compared with our observations of a very rapid inhibition helps further distinguish this free extracellular enzyme-independent and putatively direct mechanism of microbe-cathode interaction.

### M. barkeri cells are in direct contact with carbon electrodes.

**FIG 5:**
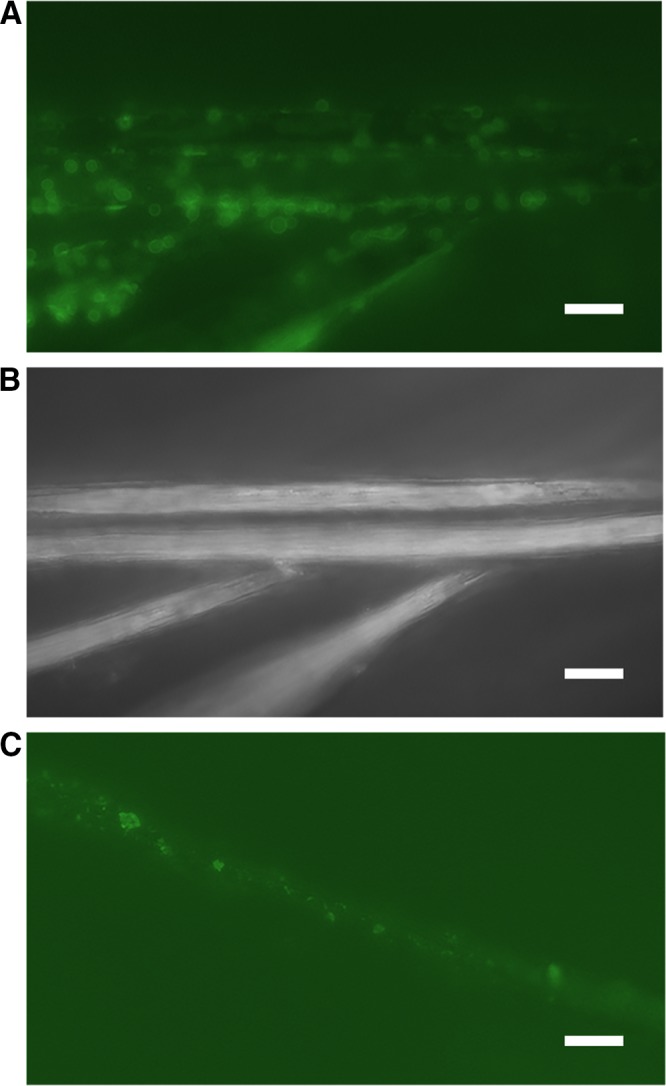
*M. barkeri* attach to carbon cloth fibers of electrode. (A and B) Experiment treated with NanoOrange protein stain in washed cell experiments to highlight protein of the cells surface layer (S-layer) in contact with the carbon fibers of the carbon cloth electrode (A), which are highlighted in a reflective light image (B). (C) NanoOrange staining of spent medium extracts highlights the attachment of proteinaceous material to carbon fibers in spent medium-only experiments. Images were taken 5 days postincubation at −500 mV. Scale bar indicates a 10 micron length.

To investigate the potential for direct cell-electrode interactions, M. barkeri cells from washed culture experiments were stained with NanoOrange and visualized by fluorescence microscopy post-H-cell inoculation and electrochemical incubations (∼5 to 7 days) (Fig. 5). Direct contact between cell surfaces and carbon cloth electrode fibers could be observed, with attached cells similar in size and shape (1- to 2-µm cocci) to previously described planktonic M. barkeri cells (26). Similar patterns of cell attachment were also observed using scanning electron microscopy (Fig. S3). In the spent medium controls, intact cells were not observed, but proteinaceous material stained with NanoOrange appeared to attach to the electrodes (Fig. 5). Combined with previous observations, these data provide evidence for potential direct cell-electrode contacts, providing further support for putatively direct and free extracellular enzyme-independent interactions.

10.1128/mBio.02448-18.3 FIG S3 Methanogen cell attachment to carbon fibers of electrode shown via scanning electron microscopy. Images taken of wild-type washed culture experiments poised at −450 mV for 5 to 7 days. Download FIG S3, DOCX file, 0.5 MB. Copyright © 2019 Rowe et al. 2019 Rowe et al. This content is distributed under the terms of the Creative Commons Attribution 4.0 International license.

## DISCUSSION

The data presented here demonstrate the ability of M. barkeri to perform electron uptake from cathodes using at least two pathways, which also allow coupling this current consumption to methane production. The higher-potential pathway (midpoint potential, −279 mV) is consistent with the mechanism recently described in Methanococcus maripaludis, where attachment of cell-derived free enzymes to carbon electrodes results in the formation of electron donors for methanogenesis (predominantly formate and hydrogen) (18). Here, we provide electrochemical (cyclic voltammetry) data that support this observation of free extracellular enzyme-mediated activity. Specifically, the similarity in redox potentials between the electrode oxidation and electrode reduction sweeps suggests a tightly coupled electron transfer event similar to those observed in thin-film or protein-film voltammetry (19, 20). This mode of electrochemical activity does not require cells to contact an electrode and seems unlikely to explain the mechanism of direct interspecies electron transfer that has been described previously in microbial consortia (17).

We also detected a previously undescribed hydrogen-independent and free extracellular enzyme-independent mechanism of methane-linked electron uptake on electrodes. The redox potential of this pathway is distinctly lower than those observed for hydrogenases. While this lower potential (electron uptake potential −484 mV) presents even more favorable energetics for hydrogen evolution, we directly tested the involvement of hydrogenases by capitalizing on the reversibility of hydrogenases using elevated hydrogen concentration as a means to inhibit hydrogen formation and drive hydrogen reduction. In addition, we performed experiments using an M. barkeri deletion mutant lacking all known methane-linked hydrogenases, which maintained this low-potential (−484 mV) electron uptake coupled to methanogenesis despite the deletion of hydrogenases. Previous work in an M. maripaludis hydrogenase deletion mutant demonstrated the ability of extracellular formate dehydrogenases to yield cathodic current through formate production (18). M. barkeri, however, has not previously been shown to grow on formate and was not capable of growth using formate under our experimental conditions. Formate addition to our electrochemical experiments resulted in neither stimulation nor inhibition of the electrochemical activity observed, confirming that formate dehydrogenases are unlikely to be playing a role (Fig. S4).

10.1128/mBio.02448-18.4 FIG S4 Addition of formate does not alter currents observed in *M. barkeri* chronoamperometry and cyclic voltammetry experiments. (A) Current generation over time monitored for *M. barkeri* growth cultures (cells plus growth medium), spent medium (no cells), and washed cells of both the wild type and a hydrogenase deletion mutant (Δ*hyd*). Electrodes were poised at −500 mV for these experiments, and formate was added from a 1 M stock to a final concentration of 2 mM at ∼115 h. (B) Cyclic voltammetry (1 mV/s) data of growth culture experiments pre- and post-formate addition. CVs were performed at ∼110 h and 120 h during the experiment. Download FIG S4, DOCX file, 0.1 MB. Copyright © 2019 Rowe et al. 2019 Rowe et al. This content is distributed under the terms of the Creative Commons Attribution 4.0 International license.

The difference in biochemistries between M. maripaludis and M. barkeri likely underlie the differences observed in the predominant mode of electron uptake observed in these electrochemical systems. M. maripaludis falls phylogenetically in the clade of methanogens without cytochromes, while M. barkeri falls within the clade containing cytochromes. Though several steps in the biochemical pathway between these clades are conserved, a few steps are distinct and result in fundamental differences in the amount of energy captured, as well as in the overall reaction rate of methane formation (6). The observed low-potential electron uptake feature described in this work may be another example of a biochemical distinction between these two groups. It is feasible that the ability to transfer an electron from a solid-phase substrate (electrode) or directly from another cell is due to a related phenomenon, as has been observed in these electrochemical systems. In this case, the −484-mV potential at which electron uptake was observed suggests an energetic advantage for cells using direct interspecies electron transfer. Obtaining electrons at a lower redox potential than hydrogen could be energetically beneficial for regenerating reduced ferredoxin required in methanogenesis, mitigating the need for consuming ion motive force. However, the question of what syntrophic metabolism would support this electron transfer is unclear, though it should be noted that ferredoxin in an intermediate formed during ethanol oxidation. Though more evidence is required to confirm yield, these data suggest a possible ecologic trade-off for methanogens using direct interspecies electron transfer compared with interspecies hydrogen transfer. As of yet, only members of the cytochrome-containing methanogen clade have been shown to participate in hydrogen/formate-independent syntrophies with organisms such as G. metallireducens. Notably, G. metallireducens lacks both the confurcating hydrogenases that have been shown to function in hydrogen-producing syntrophic fermenters (17) and a substrate-level phosphorylization system for making ATP instead of ferredoxin (27). In short, it is unclear how ferredoxin is recycled during ethanol fermentation and how overall energy conservation occurs during syntrophic ethanol oxidation in this organism.

Though this work provides support from the methanogen perspective for direct interspecies electron transfer, it also raises some questions about the potential bioenergetics and ecological trade-off for different strategies of interspecies electron transfer. Future work will continue to investigate this novel mode of electron uptake, its kinetic properties, and its biophysical basis.

## MATERIALS AND METHODS

### Culturing conditions.

The strains of Methanosarcina barkeri used have been described previously (24, 28). In brief, a background strain containing the Δ*hpt*::Φ*C31int-attP* promoter fusion was utilized for wild-type experiments, as this strain was utilized for genetic manipulations (29, 30). A hydrogenase deletion mutant with the Ech, Vhu, and Fhr hydrogenases removed was utilized for delta-hydrogenase experiments (24). Each strain was provided by William Metcalf at the University of Illinois at Urbana-Champaign. All strains were grown preelectrochemical studies in a high-salt medium using 5 mM dithiothreitol (DTT) as a reductant (26) at 30°C with 100 mM methanol as the sole methanogenic substrate (i.e., methane via methanol disproportionation). The same high-salt medium was used for electrochemical experiments, with the exception that the addition of vitamin and mineral mixes, 0.001% resazurin, 5 mM DTT, and MeOH were omitted. This medium was used in both chambers of the H-cell.

Prior to electrochemical tests, cells were grown to mid-log phase on methanol. A 10% dilution of this biomass was added to each working electrode electrochemical chamber. For growth culture incubations, 10 ml of culture was added directly to the working electrode chamber of each reactor. In the case of washed culture incubations (both open circuit and poised potential experiments), 10 ml of culture was centrifuged at 9,000 × *g* for 10 min in an anaerobic chamber. Cell pellets were resuspended in reactor medium and added to the respective H-cells. Spent medium-only experiments were performed with 10 ml of medium centrifuged at a maximum speed of 13,000 × *g* for 20 min, as filtering extracts appeared to diminish extracellular protein concentration or activity.

### Electrochemical reactors.

H-cell reactors (Adams and Chittenden Scientific Glass, Berkeley, CA) equipped with two sampling ports in the 150-ml working electrode chamber were utilized for methanogen electrochemical experiments. A Nafion 117 proton exchange membrane separated the working electrode from counter- and reference electrode processes. The membrane was secured via two o-rings and attachment of a vacuum clamp (setup described previously [18, 31]). Ethanol-treated carbon cloth attached (∼2- by 3-cm rectangle) to a titanium wire (32) was utilized for a working electrode. Titanium wire attached to a 4-cm platinum wire was utilized for the counterelectrode. Ag/AgCl reference electrodes were purchased from Basi, Inc. Reference electrodes were calibrated to a standard unused reference before each experiment and stored in a 3 M KCl solution between experiments. The headspace of H-cells was maintained in an 80%/20% N_2_/CO_2_ atmosphere during experiments. The activity of hydrogenases was tested by exchanging gas headspace with an 80%/20% H_2_/CO_2_ mixture. Using the Henrys constant (K_H_*^pc^*) of 1,300 liters · atm/mol, this gives a dissolved hydrogen concentration around 0.6 mM, though actual values may be larger due to individual H-cell overpressures.

### Electrochemical conditions.

Electrochemical experiments were performed using a CH1010 potentiostat (CHInstrument, TX). Amperometry experiments were run at poised potentials of −300 to −500 mV versus SHE. Cyclic voltammetry was run over a potential window from +200 to −800 mV using a scan rate of 1 mV/s unless otherwise specified. Unless otherwise stated, all CV experiments were run postchronoamperometry (experiments run for approximately 5 days). First derivatives were calculated using the Origin 61 software package (OriginLab, MA).

### Gas analysis.

Methane production was calculated from headspace analysis using a flame ionizing detector (FID) and thermal conductivity detector (TCD)-equipped gas chromatograph (Shimadzu gas analyzer), as previously described (33). Gas concentrations were calibrated using a 1% and 0.5% standard gas mixture (Restek, PA).

### Protein quantification.

Protein content for this work was analyzed using the NanoOrange protein quantification kit. In short, cell pellets or carbon cloth electrode samples were boiled in 0.5 M NaOH for 10 min. Dilutions were quantified to ensure sample analysis within the range of 10 ng/ml to 10 µg/ml. Fluorescence was quantified using the Opti-Fluor plate reader.

### Visualization of electrode biomass.

Electrode samples were fixed using 2.5% glutaraldehyde in a 25 HEPES buffer (pH 7.5) for 30 min. Postfixation samples received six to 10 washes with 50 mM HEPES buffer at pH 7.5. NanoOrange protein stain was used to visualize cells using fluorescence microscopy as per the manufacturer’s protocol. For scanning electron microscopy (SEM), electrodes were dehydrated in ethanol, and then critical point drying using hexamethyldisilazane (HMDS) was used prior to visualization (34).
