# Supplementary material for: Methane-Linked Mechanisms of Electron Uptake from Cathodes by Methanosarcina barkeri
Source: mBio. 2019 Mar 12;10(2):e02448-18. doi: 10.1128/mBio.02448-18 (PMC6414700; doi:10.1128/mBio.02448-18)
Supplement: FIG S1 [file mBio.02448-18-sf001.docx]

**
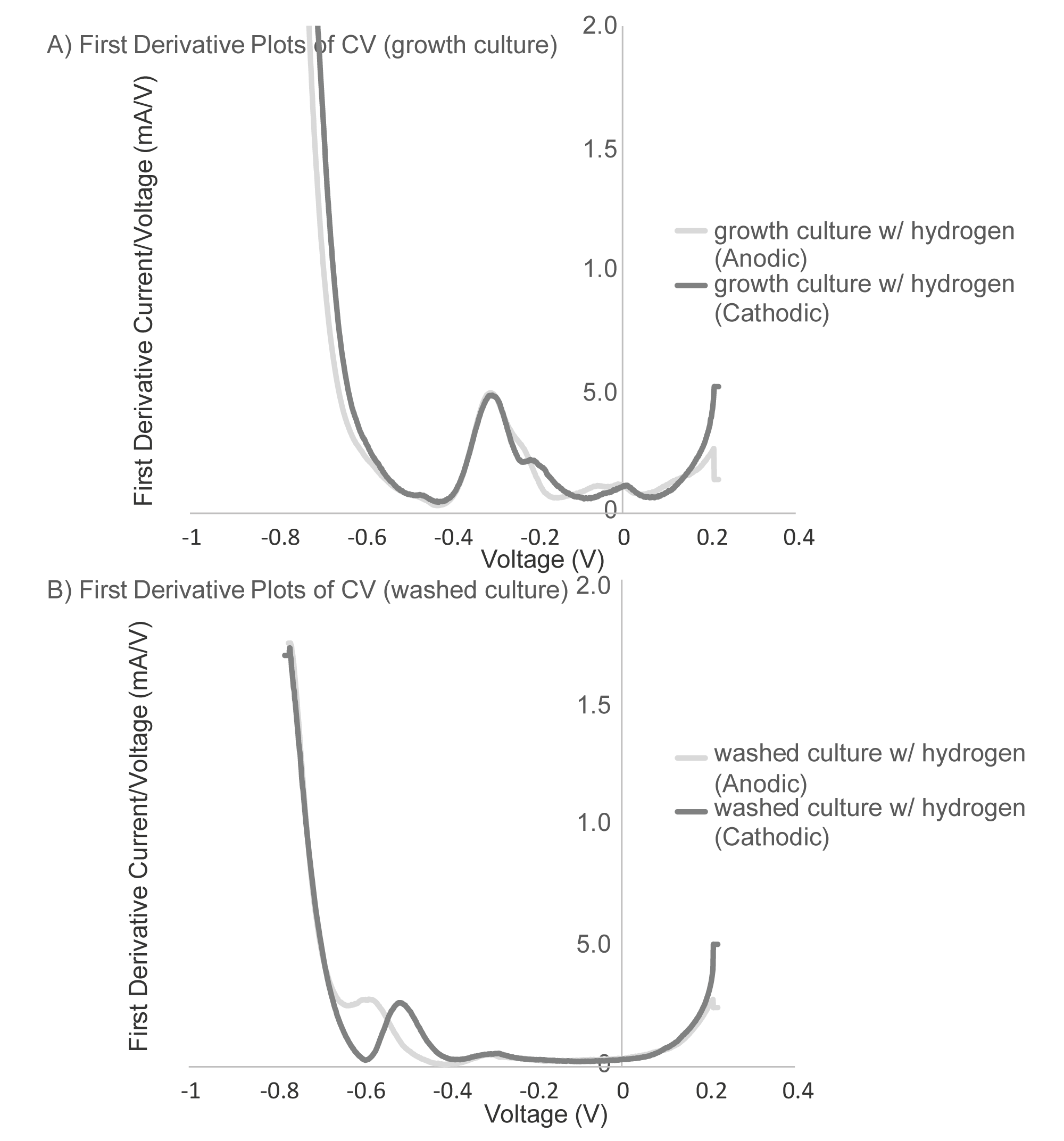
**

**Figure S1. Dominant electron uptake feature in spent media + cells experiments resemble an electrode bound enzyme process.** First derivative plots of anodic (negative to positive) and cathodic (positive to negative) sweeps from cyclic voltammetry of growth culture (A) and washed culture (B) experiments. Cyclic voltammetry data shown in Figure 2 of main text.
