# Supplementary material for: Methane-Linked Mechanisms of Electron Uptake from Cathodes by Methanosarcina barkeri
Source: mBio. 2019 Mar 12;10(2):e02448-18. doi: 10.1128/mBio.02448-18 (PMC6414700; doi:10.1128/mBio.02448-18)
Supplement: FIG S2 [file mBio.02448-18-sf002.docx]

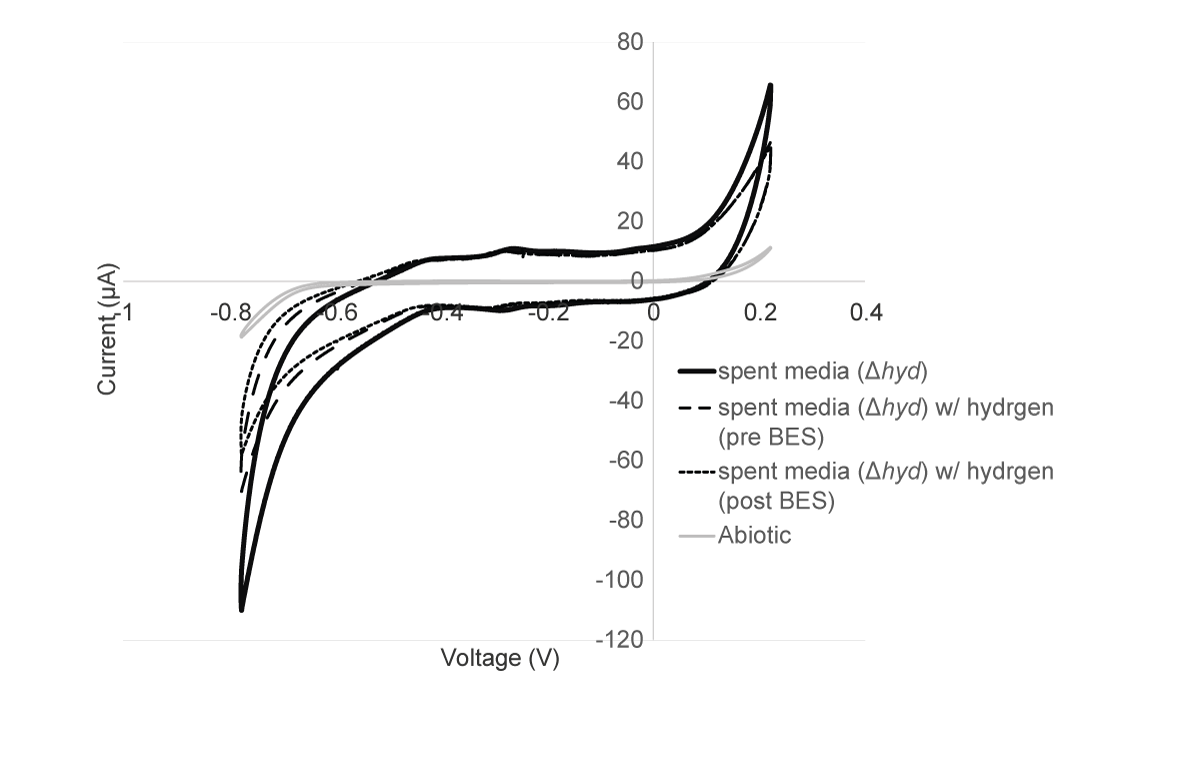


**Figure S2. Spent media of hydrogenase deletion mutant shows little to no change with addition of hydrogen and BES.** Cyclic voltammetry (1 mV/sec scan rate over a -800 to 200 mV range) showing current-voltage relationship for spent media only controls under experimental conditions with a nitrogen and carbon dioxide atmosphere (80%/20% N_2_/CO_2_), a hydrogen and carbon dioxide atmosphere (80%/20% H_2_/CO_2_) and with 7 mM BES added.
