# Supplementary material for: Methane-Linked Mechanisms of Electron Uptake from Cathodes by Methanosarcina barkeri
Source: mBio. 2019 Mar 12;10(2):e02448-18. doi: 10.1128/mBio.02448-18 (PMC6414700; doi:10.1128/mBio.02448-18)
Supplement: FIG S3 [file mBio.02448-18-sf003.docx]

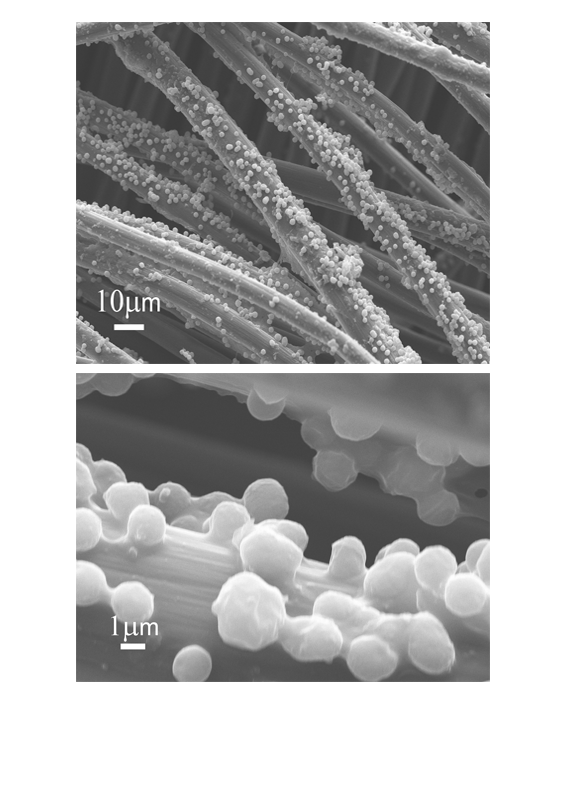


**Figure S3. Methanogen cell attachment to carbon fibers of electrode shown via scanning electron microscopy.** Images taken of wild-type washed culture experiments poised at -450 mV for 5 to 7 days.
