# Supplementary material for: Methane-Linked Mechanisms of Electron Uptake from Cathodes by Methanosarcina barkeri
Source: mBio. 2019 Mar 12;10(2):e02448-18. doi: 10.1128/mBio.02448-18 (PMC6414700; doi:10.1128/mBio.02448-18)
Supplement: FIG S4 [file mBio.02448-18-sf004.docx]

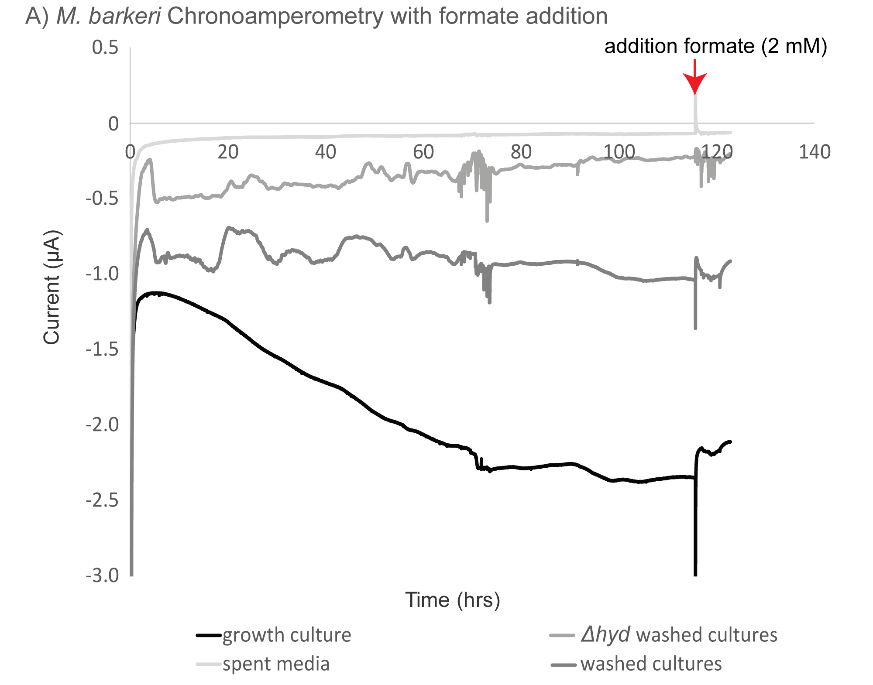

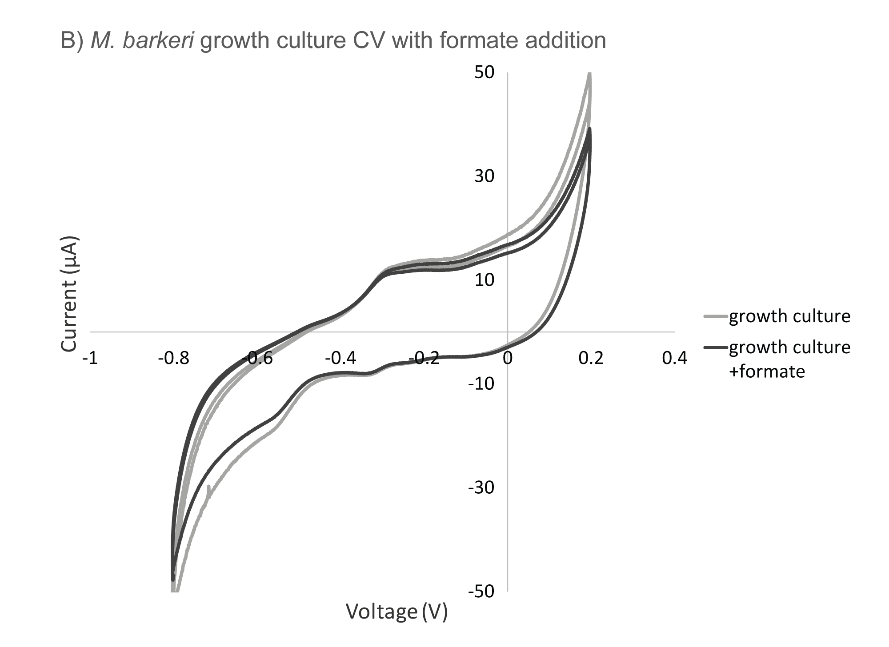


**Figure S4. Addition of formate does not alter currents observed in *M. barkeri* chronoamperometry and cyclic voltammetry experiments.** (A) Current generation over time monitored for M. barkeri growth cultures (cells + growth media), spent media (no cells), and washed cells of both wild type and a hydrogenase deletion mutant (Δ*hyd*). Electrodes poised at -500 mV for these experiments, and formate was added from a 1M stock to a final concentration of 2 mM at ~ 115 hours. (B) Cyclic voltammetry (1 mV/sec) data of growth culture experiments pre and post formate addition. CV’s performed at ~110 hrs and 120 hrs during the experiment.
